# Supplementary material for: Supplementation of Weizmannia coagulans BC2000 and Ellagic Acid Inhibits High-Fat-Induced Hypercholesterolemia by Promoting Liver Primary Bile Acid Biosynthesis and Intestinal Cholesterol Excretion in Mice
Source: Microorganisms. 2023 Jan 19;11(2):264. doi: 10.3390/microorganisms11020264 (PMC9964488; doi:10.3390/microorganisms11020264)
Supplement: Supplementary file 1 [file microorganisms-11-00264-s001.zip › microorganisms-2177892-Supplementary materials.pdf]

Supplementary materials

Methods

Method S1 Preparation of animal feeds

Lyophilized *Weizmannia coagulans* powder, Mineral mix S10026, and Vitamin mix V10001 were pre-mixed with maltodextrin by grinding in a large mortar for feed with lard. Liquefied lard is mechanically mixed with Casein to form a homogeneous pellet. These two prepared premixes were gradually added to the other feed components (Table S1, purchased from Guangdong Medical Laboratory Animal Centre, China) during mixing in a mixer (Lihongl587923 China) for 12 h (6 h counter-clockwise, 6 h clockwise). The homogeneously mixed feed is processed into stick-shaped pellets using a pellet mill (Limanman180 China). They were dispensed into ziplock bags and stored at -20°C until use. Low-fat feeds are processed the same way, except no pre-mixing of lard is done.

Method S2 Analysis of EA and urolithins by UPLC-ESI-QTOF-MS/MS

Metabolites analysis was carried out using an Agilent 1290 Infinity UPLC system connected to a 6530B Accurate-Mass quadrupole TOF mass spectrometer (Agilent Technologies, Waldbronn, Germany) using an electrospray interface and Jet Stream technology. Separation was achieved on a reverse phase Poroshe ll 120 EC-C18 column (3 × 100 mm, 2.7 µm; Agilent) operating at 30°C. The mobile phases were water: formic acid (99.9:0.1 v/v; phase A) and CAN: formic acid (99.9:0.1 v/v; phase B). The gradient program was as follows: 0–3 min, 5–15% B; 3–11 min, 15–30% B;11–15 min, 30–50% B, 15–21 min, 50–90% B. Finally, the B content was decreased to the initial conditions (5%) in 1 min and the column was equilibrated for 5 min. The flow rate was set constant at 0.4 mL/min and the injection volume was 3 µL. The optimal conditions of the electrospray interface were as follows: gas temperature 280°C, drying gas 9 L/min, nebulizer 45 psi, sheath gas temperature 400°C, sheath gas flow12 L/min. Spectra were acquired in single MS mode with an m/z range of 100–1100, negative polarity, and an acquisition rate of 1.5 spectra/s. MS/MS product ion spectra were collected at an m/z range of 50–800 using a retention time window of 1 min, collision energy of 20 V and an acquisition rate of 4 spectra/s. Targeted MS/MS experiments provided fragmentation information, providing additional confidence in the compound identification process.

Method S3 Untargeted metabolomics of plasma by UPLC-QTOF-MS

Chromatographic Conditions: The plasma untargeted metabolomics analysis was performed using Agilent 1290 Infinity UPLC system (Agilent Technologies, Palo Alto, CA, United States). ZORBAX Eclipse Plus C18 RRHD columns (2.1 × 150 mm, 1.8 μm) with a temperature that was maintained at 35°C were used in this analysis. The mobile phase was composed of A (0.1% formic acid in water) and B (0.1% formic acid in acetonitrile). The flow rate of the mobile phase was set at 0.4 ml/min. The linear gradient elution program was set as follows: at 0–2 min, 98% A and 2% B; 2–9 min, 98% A and 2% B; 9–15 min, 55% A and 45% B; 15–22 min, 30% A and 70% B; 22–24 min, 2% A and 98% B; and 24–26 min, 98% A and 2% B.

Mass Spectrometry Conditions: The metabolomics analysis was performed using a 6530B QTOF mass spectrometer (Agilent Technologies, Palo Alto, CA, United States), which was equipped with an electrospray ionization (ESI) source. The positive mode was used to collect the data from 50 to 1000 m/z at a rate of 1 spectra/s. The optimal conditions were set as follows: fragmentation voltage: 135 V; the skimmer voltage: 65 V; capillary voltage: ESI +: 4.0 kv, ESI-: 3.5 kV; desolvation gas flow: nitrogen, 10 L/min, 350°C, atomizer pressure: 45 psi.

Tables

Table S1 Feed formula (g/kg)

Composition	LFD	HFD	EA	EABC77	EABC2000
Casein	189.6	231	231	231	231
L-Cystine	2.8	3	3	3	3
Corn Starch	479.8	83.9	83.6	83.5	83.5
Maltodextrin	118.5	115	115	115	115
Sucrose	65.2	199.3	199.3	199.3	199.3
Cellulose	47.4	58	58	58	58
Soybean oil	23.7	29	29	29	29
Lard	19	204.7	204.7	204.7	204.7
Mineral Mix	9.5	12	12	12	12
Dicalcium Phosphate	12.3	15	15	15	15
Calcium Carbonate	5.2	6.3	6.3	6.3	6.3
Potassium Citrate·H_2O	15.6	19	19	19	19
Vitamin Mix	9.5	12	12	12	12
Choline Bitartrate	1.9	2	2	2	2
Cholesterol	0	9.8	9.8	9.8	9.8
Ellagic acid	0	0	0.3	0.3	0.3
Probiotic powder	0	0	0	0.1	0.1
Total (g)	1000	1000	1000	1000	1000
Calories (kcal/g)					
Protein	20%	20%	20%	20%	20%
Carbohydrate	70%	35%	35%	35%	35%
Fat	10%	45%	45%	45%	45%
Total	100	100	100	100	100

Note: LFD: low-fat-diet group; HFD: high-fat-diet group; EA: ellagic acid intervention group; EABC77: EA + *Weizmannia coagulans* BC77 intervention group; EABC2000: EA + *Weizmannia coagulans* BC2000 intervention group.

Figures

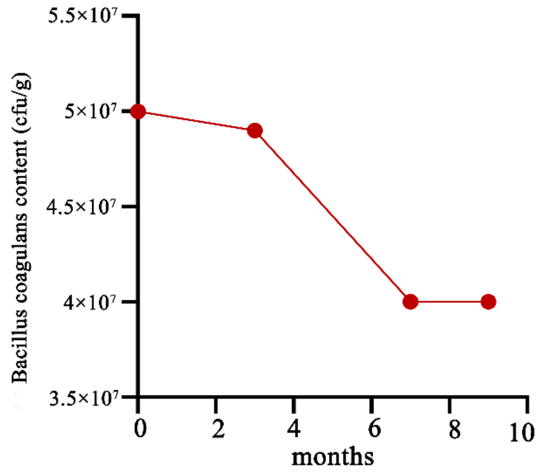

Figure S1. Stability of *Weizmannia coagulans* in probiotic nuts at different time points.

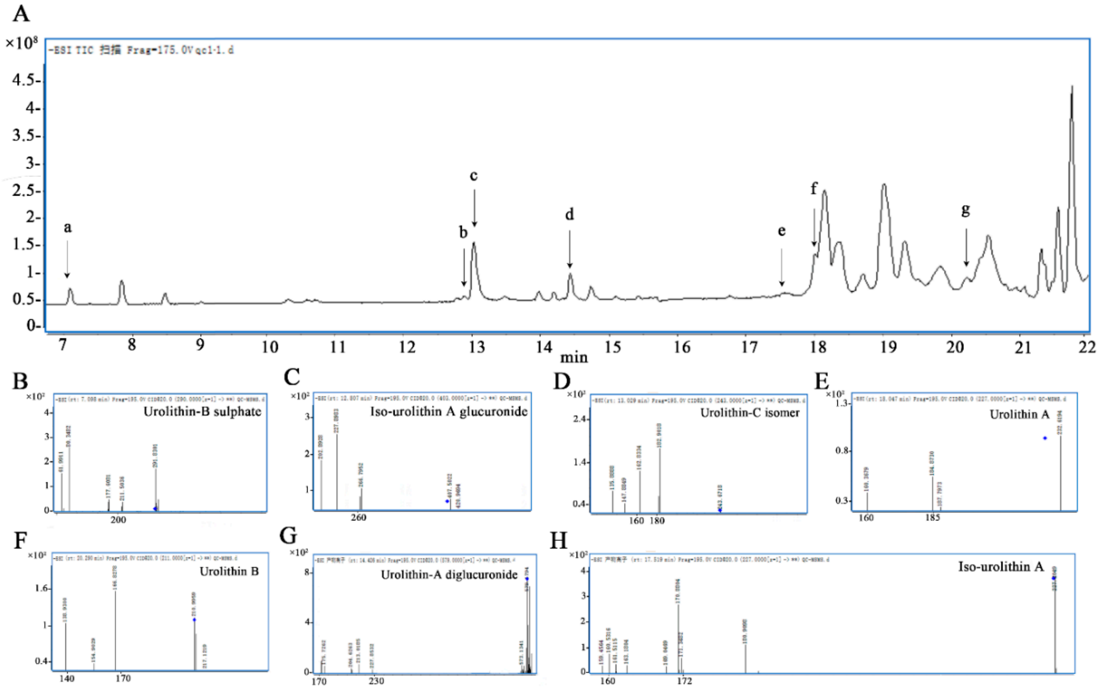

Figure S2. Effect *Weizmannia coagulans* on EA-derived metabolites in the mouse liver. (**A**) Chromatogram of ellagic acid metabolites obtained by UPLC-ESI-QTOF-MS/MS; (**B**) MS/MS spectra of Urolithin-B sulfate; (**C**) MS/MS spectra of Iso-urolithin A glucuronide; (**D**) MS/MS spectra of Urolithin-C isomer; (**E**) MS/MS spectra of Urolithin A; (**F**) MS/MS spectra of Urolithin B; (**G**) MS/MS spectra of Urolithin-A diglucuronide; (**H**) MS/MS spectra of Isurolithin A; "a" indicates Urolithin-B sulphate; "b" indicates Iso-urolithin A glucuronide; "c" indicates Urolithin-C isomer; "d" indicates Urolithin-A diglucuronide; "e" indicates Iso-urolithin A; "f" indicates Urolithin A; "g" indicates Urolithin B. N = 6 in each group.
